# Impact of Powdered Tart Cherry Supplementation on Performance Recovery Following Repeated Sprint Exercise

**DOI:** 10.3390/nu18030443

**Published:** 2026-01-29

**Authors:** Anthony M. Hagele, Kyle S. Levers, Kevin F. Holley, Alex C. Schrautemeier, Joesi M. Krieger, Joshua M. Iannotti, Connor J. Gaige, Ralf Jäger, Chad M. Kerksick

**Affiliations:** 1 Exercise and Performance Nutrition Laboratory, Lindenwood University, 209 South Kingshighway, St. Charles, MO 63301, USA; ahagele@lindenwood.edu (A.M.H.); kholley@lindenwood.edu (K.F.H.); jmorey@lindenwood.edu (J.M.K.); joshuamiannotti@gmail.com (J.M.I.); 2 Metabolism and Exercise Testing Laboratory, Milken Institute School of Public Health, George Washington University, Washington, DC 20052, USA; klevers@gwu.edu; 3 Toriola Laboratory, Department of Surgery, Washington University, St. Louis, MO 63110, USA; schrautemeier.alex@gmail.com; 4 Dominantly Inherited Alzheimer Network Trials Unit, Department of Neurology, Washington University School of Medicine, St. Louis, MO 63310, USA; gaige@wustl.edu; 5 Increnovo LLC, Whitefish Bay, WI 53217, USA

**Keywords:** tart cherry, dietary supplement, repeated sprint exercise, exercise performance

## Abstract

Background: Due to its high polyphenol content and purported capability to mitigate post-exercise muscle soreness and promote recovery, tart cherry (TC) supplementation has been proposed to enhance recovery and athletic performance. This study examined the effects of powdered TC supplementation on various recovery and performance metrics following a repeated sprint exercise protocol in physically active young adults. Methods: 40 (18 M, 22 F) healthy, active participants (24.6 ± 5.5 yrs, 171.5 ± 11 cm, 71.7 ± 14.5 kg, 24.2 ± 3.1 kg·m^−2^) participated in this randomized, double-blind, placebo-controlled, parallel study design. Placebo (PLA) or powdered TC supplementation (500 mg/day) occurred for ten days: seven days prior to, day of, and two days following repeated sprints (15 × 30 m with 1 min rest between sprints). Performance was assessed via the countermovement jump, isometric mid-thigh pull, isokinetic knee extension, and the Wingate anaerobic test. Recovery was evaluated using visual analog scales for soreness, recovery, and readiness to train. Muscle damage was evaluated using creatine kinase. These measures were evaluated at baseline, and at 1 h, 24 h, and 48 h post-exercise. Results: Significant main effects of time were observed with recovery VAS (*p* < 0.001), readiness to train VAS (*p* < 0.001), and jump height (*p* = 0.014) experiencing similar reductions, while soreness VAS (*p* < 0.001) and creatine kinase (*p* = 0.05) experienced similar increases in response to the repeated sprint protocol and supplementation. Across all measurements, no significant group × time differences were observed for jump height (PLA:−6.7 ± 10.4% vs. TC: −11.0 ± 17.9%, *p* = 0.608), peak propulsive force (PLA: 0.3 ± 4.6% vs. TC: 2.2 ± 7.4%, *p* = 0.194), knee extension peak torque at 180°/s (PLA: 10.5 ± 73.5% vs. TC: −1.04 ± 49.6%, *p* = 0.335), readiness to train VAS (PLA: −23.0 ± 19.2% vs. TC: −14.7 ± 20.2%, *p* = 0.401), soreness VAS (PLA: 250 ± 323% vs. TC: 261 ± 432%, *p* = 0.838), recovery VAS (PLA: −24.6 ± 17.9% vs. TC: −8.2 ± 40.5%, *p* = 0.251), and creatine kinase (PLA: 22.8 ± 35.5% vs. TC: 90.4 ± 225.6%, *p* = 0.31). Conclusions: A single bout of repeated sprints was responsible for significant reductions in jump height, peak propulsive force, peak torque, and perceived readiness, while perceived soreness, myoglobin, and creatine kinase were significantly increased. Ten days of TC supplementation did not impact any change beyond what was observed in PLA for markers of recovery, readiness, soreness, exercise performance, and markers of muscle damage.

## 1. Introduction

High volume repetition of intense anaerobic performance bouts is expressed across a multitude of athletic endeavors through several modes of gameplay. Ground-based athletic competition such as soccer, football, rugby, lacrosse, basketball, and tennis, impart high-energy demand consisting of high volumes of concentric, eccentric, and isometric muscle actions, which stress both the mechanical and metabolic systems [1,2]. Exercise-induced muscle damage (EIMD) commonly results from these extended duration, perhaps unfamiliar, events where eccentric-focused mechanical stress is carried out at a high metabolic intensity [3,4,5,6,7]. EIMD typically diminishes subsequent athletic performance via symptoms such as delayed-onset muscle soreness (DOMS), edema, compromised joint range of motion, impaired neuromuscular strength and power, and inflammation [3,7,8,9].

Acute bouts of strenuous athletic performance commonly replicated in the literature using a single large volume, high intensity anaerobic event facilitate EIMD, characterized by underlying mechanical muscle damage, oxidative damage, and inflammation [10,11,12,13,14]. Specifically, the repetitive load-induced stress results in muscular injury via ultrastructural disruptions [15,16,17] and the subsequent release of intramuscular proteins into systemic circulation [18,19] that ultimately kickstarts the muscular repair sequence of degeneration, inflammation, regeneration, and fibrosis [15,20]. EIMD following damaging exercise is a consequence of high nociceptor and mechanoreceptor sensitivity to the potent chemicals and by-products released during this muscular degeneration [3,9,21]. In conjunction with the damage-inducing inflammatory process, higher volumes of repetitive muscular stress may also overwhelm the capacity of the endogenous antioxidant system to balance free radical production [22,23]. Collectively, symptoms of EIMD alongside the underlying repair and recovery process will prolong return to peak performance, impairing subsequently planned training and competition.

For various reasons, nutritional research over the last 10–15 years has demonstrated increased emphasis toward the study of phytochemical-containing fruits and other functional foods that seem to provide a beneficial anti-inflammatory and antioxidant effect [24,25]. Phenolic compounds, such as flavonoids and anthocyanins, may act to support endogenous antioxidant systems to expedite exercise recovery [22,26]. Of the wide variety of antioxidant and polyphenol-containing functional foods, tart cherry (TC) supplement formulations have received attention within the clinical and sports science literature due to their high anthocyanin content [27], which has demonstrated consistent benefit as a naturally occurring intervention to aid improvement in health [28,29,30], inflammatory-related disease states [29,31,32], and sleep quality [33,34]. To this point, the initial clinical nutrition support realized from TC supplementation spurred sport nutrition researchers to use juice, concentrate, gel, and powder formulations to support the performance of endogenous physiological systems in managing muscle damage, oxidative stress, and inflammation following challenging exercise scenarios.

For example, TC supplementation surrounding various bouts of resistance-based exercise in college-aged recreationally active and trained males has demonstrated expedited muscular force production recovery compared to placebo (PLA) [18,24,35,36] across multiple TC formulations, with less strength recovery impact in female [37] and non-resistance trained populations [38]. Irrespective of the formulation, subject sex, and training experience, short-term TC supplementation has shown limited or inconsistent effects on post-exercise muscle tenderness and swelling [11,24,36,37,38], joint range of motion capacity [11,24,38], indices of muscle damage [11,18], neuromuscular power performance [36,37], and quadricep muscular activation [37] surrounding single joint open kinetic chain resistive movements such as knee extensions. While influence on oxidative damage following these types of movements with TC appears incongruent, TC supplementation surrounding high-volume closed-kinetic chain movements has demonstrated some promise attenuating post-exercise muscular strength decrement [35], muscle tenderness, or perceptions of pain [35,39], while remaining inconclusive regarding damage and oxidative stress indices [35,39].

High-volume intermittent work, such as repeated sprint exercise, provides a more ecologically valid model for evaluating TC supplementation under anaerobic, sport-relevant conditions. Unlike the inconsistency observed across the scientific literature involving different types of resistance exercise, short-term TC supplementation collectively demonstrates attenuated perceptions of post-exercise muscle soreness [4,40,41,42] and pain pressure threshold [4] while facilitating greater muscular force production [40,41,42] and measures of neuromuscular power [4,40,41,42] surrounding various iterations of repeated sprint-interval (RSI) challenges. With further consistency, the current literature examining repeated sprints exhibits the limited impact of TC supplementation after strenuous exercise on indices of muscle damage [4,40,41] and inflammation [4,41].

Despite growing interest, the TC literature remains limited by the exclusion of females, minimal insight into changes related to hormone status and inflammation, and inconsistent use of exercise protocols that offer limited translation to sporting activity. For these reasons, the primary aim of the current study was to determine if short-term (10 days) ingestion of a powdered TC formulation surrounding a bout of repeated sprints would impact recovery, circulating concentrations of anabolic and catabolic hormones, muscle damage, oxidative stress, and inflammation connected to recovery, muscle damage, and inflammation, perception of subsequent performance readiness, and ensuing exercise performance in a larger pool of recreationally trained males and females. We hypothesized that recreationally active participants will be better prepared for successive bouts of physical performance via reduced muscle soreness and damage when supplementing with a powdered TC formulation surrounding a demanding repeated sprint protocol.

## 2. Materials and Methods

### 2.1. Experimental Design

**Figure 1:**
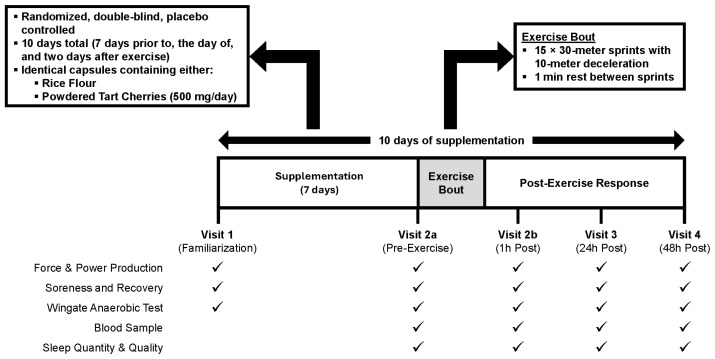
Overview of research design.

The study utilized a randomized, double-blind, placebo-controlled, parallel group study design (Figure 1). Prior to data collection, potential participants reviewed the protocol and provided written informed consent via an IRB-approved consent form. Healthy male and female participants (*n* = 40) who regularly participated in recreational sports or high-intensity, multi-modal resistance-based exercise were included. Regular participation was defined as exercising at least four days per week, with a minimum of two days involving high-intensity exercise, for the past six months, as determined by the International Physical Activity Questionnaire. Participants complete four laboratory visits. Visit 1 involved initial screening, assessment of height, body mass, and body composition, and concluded with familiarization with the testing procedures. Once eligibility was confirmed, participants were randomized by biological sex and baseline fat-free mass to ingest either PLA (rice flour) or powdered tart cherries (ADSO Naturals, Bangalore, India). Participants consumed the supplements over a 10-day period, with doses 1–7 taken daily before returning to the lab for Visit 2. Dose 8 was taken on the morning of Visit 2, dose 9 on the morning of Visit 3, and dose 10 on the morning of Visit 4. Body mass, resting blood pressure, and resting heart rate were assessed during all visits.

Visit 2 served as the baseline testing session and the start of the post-exercise recovery period. During Visit 2, a venous blood sample was taken to measure baseline markers of health, muscle damage, oxidative stress, inflammation, testosterone, and cortisol. Participants completed visual analog scales to provide perceived levels of recovery, soreness, and readiness to exercise before completing a standardized warm-up. Performance testing was completed in a fixed order: (1) countermovement jumps, (2) isometric voluntary contractions, (3) isokinetic dynamometry using the knee extensors, and (4) a Wingate anaerobic test. This sequence was identical for all participants and across all applicable testing timepoints. Standardized rest periods were provided between performance assessments to minimize fatigue carryover. Participants rested for approximately three minutes between each assessment. Baseline assessments were completed prior to the initiation of the repeated sprint protocol. The repeated sprint protocol consisted of 15 × 30 m maximal sprints performed on an environmentally controlled indoor running track, with standardized rest periods between sprints, as described below. Participants remained in the laboratory for one hour after completion of the sprint protocol to provide an additional blood sample and complete all assessments.

Blood sample collection and performance assessments were then repeated during subsequent laboratory visits that occurred 24 h (Visit 3) and 48 h (Visit 4) after completion of the sprint protocol. As such and for clarity throughout the manuscript, data collection timepoints are referred to as Baseline, 1 h Post, 24 h Post, and 48 h Post, which respectively correspond with Visits 2, 3, 4, and 5 as described herein. Each participant completed all laboratory visits at the same time of day (±1 h), with all study visits commencing between 0600 and 1000 h. Prior to each study visit, participants observed an overnight fast for 8–10 h with no caffeine or nicotine for 12 h prior. During laboratory visits, participants were permitted to consume water ad libitum to maintain hydration; however, no calorie containing beverages or food were allowed during the exercise testing repeated sprint exercise bout or rest periods. No vigorous exercise was permitted 48 h before, and only light physical activity was permitted 24 h before Visit 2. No exercise was permitted between Visits 2 and 4. Participants were further instructed to refrain from using percussive or compression devices, hot or cold showers or baths, foam rolling, or consuming any prescription or over-the-counter anti-inflammatory medications throughout the study to prevent interference with recovery and performance outcomes. The study was approved by the Institutional Review Board of Lindenwood University (IRB-23-11, approval date: 27 January 2023) and was conducted in accordance with the Declaration of Helsinki. The study was prospectively registered at ClinicalTrials.gov (identifier: NCT06122038).

### 2.2. Study Participants

Healthy, recreationally active males (*n* = 18) and females (*n* = 22) completed the entire study protocol and were included in the final analyses. To be eligible, participants were required to be engaged in at least four days of exercise per week, with a minimum of two high-intensity exercise sessions weekly for at least six months prior to the study. High-intensity exercise was defined as reporting participation in at least two days per week of vigorous-intensity physical activity as indicated on the International Physical Activity Questionnaire—Short Form (IPAQ-SF). Participants were required to be between 20 and 35 years of age and have a body mass index (BMI) of 18.0–30.0 kg/m^2^. Individuals with a BMI greater than 30.0 kg/m2 were accepted if their body fat percentage was less than 27.5% for males and 32.5% for females.

All participants were free from known cardiovascular, metabolic, renal, hepatic, or neurological disease, and were non-smokers not currently using any medications or supplements known to affect inflammation, recovery, or performance. Participants were not pregnant, lactating, or following any weight-loss protocols in the 30 days prior to enrollment. A detailed health history questionnaire was used to screen exclusion criteria, and participants were excluded if any condition was present that could compromise safety or study outcomes. Participants were instructed to discontinue all ergogenic nutritional supplements (e.g., creatine monohydrate, β-alanine) and supplements known or purported to impact muscle repair or recovery (e.g., antioxidants, creatine, HMB, curcumin, turmeric, Vitamin D, TC) for 30 days prior to participation.

### 2.3. Anthropometric, Hemodynamic, and Body Composition Assessments

During Visit 1, participants’ height was measured without shoes to the nearest 0.5 cm using a wall-mounted stadiometer (HR-200, Tanita Corp, Inc., Tokyo, Japan). Body mass was recorded to the nearest 0.1 kg during each study visit with a self-calibrating digital scale (Tanita BWB-627A, Tokyo, Japan). Resting heart rate and blood pressure were measured during each visit using an automated blood pressure cuff (3 Series^®^ Upper Arm Blood Pressure Monitor, OMRON Healthcare, Kyoto, Japan) after a 5 min seated period. Body composition, including body-fat percentage, skeletal muscle mass, and total body water, was assessed using a bioelectrical impedance analyzer (BIA; InBody 570, InBody USA, Cerritos, CA, USA). Participants removed their socks, emptied pockets, and removed heavy clothing or accessories before each scan, and wiped their hands and feet using wipes provided by the manufacturer (Inbody USA, Cerritos, CA, USA).

### 2.4. Supplementation

Following the initial familiarization visit, participants consumed their assigned supplement daily for a 10-day period. Doses 1 through 7 were taken daily leading up to Visit 2, which coincided with the repeated sprint protocol. Dose 8 was consumed on the morning of Visit 2, dose 9 on the morning of Visit 3, and dose 10 on the morning of Visit 4. Participants were instructed to take one capsule of their assigned supplement at the same time each day with their first meal, ensuring to include fats, proteins, and carbohydrates in the meal. The experimental supplement consisted of 500 mg of powdered tart cherries (TCs) (ADSO Naturals, Bangalore, India) in capsule form. The PLA group received rice flour. Total phenolic content of the powdered TC was 1.16% *w*/*w,* resulting in each 500 mg delivering a phenolic dose of 5.8 milligrams. Each PLA dose delivered approximately 0.4 g carbohydrates and 7.7 kJ for a total anticipated delivery of 4 g of carbohydrate and 76.6 kJ. Randomization and allocation concealment were maintained throughout the study. Investigators and participants remained blinded to treatment assignment until all data collection and primary analyses were completed. To ensure integrity of blinding, all capsules were non-transparent, the same color, size, and smell. To assess compliance, participants completed a daily diary documenting their supplement intake and any adverse events.

### 2.5. Repeated Sprint Exercise Bout

Participants completed the repeated sprint exercise bout following the methods of Howatson and Malik [43,44] on an environmentally controlled indoor running track marked with cones and lighted timing gates (RM-510, Arena Gear, McKinney, TX, USA). A 30 m sprint section was marked off along with a 10 m deceleration zone. Participants completed three practice sprints at 60%, 80%, and 100% perceived speed with one-minute rest between sprints. After practice sprints were completed, a three-minute break was observed. Participants then stood 12 inches from the start line to avoid premature triggering of the timing system and completed 15 separate 30 m sprints with one minute rest between each sprint. All efforts were completed using a maximal effort, and participants came to a complete stop within the 10 m deceleration zone. Strong verbal encouragement was provided throughout each sprint. Time to complete each sprint was recorded.

### 2.6. Performance Testing

#### 2.6.1. Countermovement Jumps

Countermovement jumps (CMJs) were used to assess lower-body power. Testing was conducted using a pair of force plates (Hawkin Dynamics, Westbrook, ME, USA). Participants stood with one foot in the middle of each force plate with their feet shoulder-width apart while keeping their hands on their hips throughout the movement to eliminate the influence of arm swing. All participants stood in the same direction and general orientation for all jumps in the study protocol. They initiated the movement by bending at the knees and hips to a self-selected depth before jumping as high as possible. Each participant performed three maximal effort jumps with 60 s of rest between attempts. The force plates captured ground reaction forces in real-time, which were analyzed in real-time. The highest jump height and peak propulsive force output was highlighted by the software and used for statistical analysis. CMJ testing was performed at baseline, immediately post-exercise, and at 24 and 48 h post-exercise to assess recovery dynamics. A standardized rest interval of three minutes was provided between performance testing protocols.

#### 2.6.2. Isometric Mid-Thigh Pull

The isometric mid-thigh pull (IMTP) was conducted to assess maximal isometric strength according to previously described procedures [45]. Participants performed the IMTP using a fixed bar in a custom rig attached to dual force plates (PASPORT Force Platform, PASCO Scientific, Roseville, CA, USA). Participants were instructed to stand on the force plates in the same direction with one foot in the center of each force plate and position the bar at mid-thigh height while maintaining a knee angle of approximately 125° and a torso angle of 145°, with a neutral spine and hips. After positioning, participants were instructed to pull the bar upward as hard and as fast as possible for five seconds, aiming to generate maximal force against the immovable bar. Verbal encouragement was provided throughout the test to maximize effort. Three attempts were performed at each timepoint, with one minute of rest between attempts. Peak force was recorded during each trial, with the highest value being used for analysis. IMTP testing was conducted at baseline, immediately post-exercise, and at 24 and 48 h post-exercise.

#### 2.6.3. Wingate Anaerobic Test

The Wingate anaerobic test was used to evaluate anaerobic capacity. The test was performed using a cycle ergometer (Lode Excalibur Sport, Groningen, The Netherlands). Participants began with a standardized warm-up consisting of one minute of cycling at a self-selected pace with no resistance to the pedals. After the warm-up, participants were instructed to pedal maximally for 30 s against a resistance set to 7.5% of their body mass. Throughout the test, participants were given strong verbal encouragement to maintain maximal effort. The Lode Ergometer Manager (LEM 10, Lode, Groningen, The Netherlands) recorded peak power, mean power, relative peak power, relative mean power, and total work. The Wingate test was administered at baseline, immediately post-exercise, and at 24 and 48 h post-exercise.

#### 2.6.4. Isokinetic Dynamometry

Isokinetic knee extension strength of the dominant leg was assessed using the Biodex System 3 dynamometer (Biodex Medical Systems, Shirley, NY, USA). While participants were asked to flex and extend their knee as forcefully as possible throughout each repetition, only the extension data was used as part of this study protocol. Participants were seated in the dynamometer with their hips and knees flexed at approximately 90°, and the dynamometer’s axis of rotation aligned with the lateral femoral epicondyle of the dominant leg. Straps were used to secure the thigh, pelvis, and torso to minimize extraneous movement during testing. Participants performed maximal voluntary contractions at three angular velocities: 60°/s × 5 repetitions, 180°/s × 10 repetitions, and 300°/s × 10 repetitions. Two minutes of rest were observed between each set. For each speed, participants were instructed to extend their knee as forcefully and quickly as possible. Verbal encouragement was provided to ensure maximal effort during each contraction. Peak torque values were recorded for each speed and analyzed. Testing was conducted at baseline, immediately post-exercise, and at 24 and 48 h post-exercise to evaluate muscle performance and recovery.

### 2.7. Blood Collection and Biomarker Analysis

Venous blood samples were collected from participants at baseline, immediately post-exercise, and at 24 and 48 h post-exercise. Blood was drawn from an antecubital vein using a sterile, single-stick phlebotomy technique, with samples collected into EDTA-coated tubes (plasma) and serum-separating tubes (SSTs; serum). Within 60 min of collection, samples were centrifuged at 1500× *g* for 20 min at 4 °C using a refrigerated centrifuge (MegaFuge XFR, Thermo Fisher Scientific, Waltham, MA, USA). Plasma and serum were separated and aliquoted into 500 µL portions, which were then frozen at −80 °C until subsequent analysis. Biomarker analyses were performed using commercially available enzyme-linked immunosorbent assays (ELISAs). Creatine kinase (CK) and myoglobin (ELH-Myoglobin ELISA kit, RayBiotech, Peachtree Corners, GA, USA) were analyzed as indicators of muscle damage. To evaluate oxidate stress, plasma concentrations of malondialdehyde (MDA; ab118970 ELISA kit, Abcam, Waltham, MA, USA) were analyzed as a marker of lipid peroxidation. Inflammatory cytokines were assessed to capture the inflammatory response to exercise, including plasma concentrations of tumor necrosis factor-alpha (TNF-α; ELG-TNFa ELISA Kit, RayBiotech) and Interleukin-6 (IL-6; ELH-IL6 ELISA kit, RayBiotech). Serum concentrations of total testosterone (EIA2924R ELISA kit, DRG International, Springfield, NJ, USA) and cortisol (EIA18817R ELISA kit, DRG International) were measured to assess hormonal responses to exercise and recovery. Total testosterone concentrations were analyzed as an indicator of anabolic potential, while cortisol concentrations were measured to evaluate the catabolic response to stress. The testosterone-to-cortisol ratio was calculated to provide insight into the anabolic–catabolic balance during recovery. Comprehensive metabolic panels (albumin, albumin/globulin ratio [calculated], alkaline phosphatase, alanine aminotransferase [ALT], aspartate aminotransferase [AST], blood urea nitrogen [BUN], BUN/creatinine ratio [calculated], calcium, carbon dioxide, chloride, creatinine with estimated GFR, globulin, glucose, potassium, sodium, total bilirubin, and total protein), complete blood counts (red blood cell count, white blood cell count, platelet count, hemoglobin, hematocrit, red blood cell dimension width [RDW], mean corpuscle volume [MCV], mean corpuscle hemoglobin [MCH], mean corpuscle hemoglobin content [MCHC], neutrophil % and cell count, lymphocytes % and cell count, monocytes % and cell count, eosinophils % and cell count, and basophils % and cell count [granulocytes → neutrophils, eosinophils, basophils]) and serum uric acid concentrations were analyzed by Quest Diagnostics using standard clinical laboratory procedures.

### 2.8. Pain Pressure Tolerance (PPT)

Pain pressure tolerance (PPT) was assessed using a handheld algometer (Pain Test™ FPX 10, Wagner Instruments, Greenwich, CT, USA) applied to the vastus lateralis (VL) of the dominant leg in accordance with previously published procedures [46]. The algometer was applied perpendicular to the skin at a constant rate of pressure increase (approximately 1 N/s). To ensure consistency, the application site was standardized as the midpoint between the greater trochanter and lateral epicondyle of the femur, with the location confirmed through manual palpation of the VL. The site was marked prior to the exercise protocol, and marks were maintained by the participant throughout their involvement in the study to ensure consistent placement during follow-up assessments. Participants were instructed to indicate when the sensation transitioned from pressure to pain, at which point the applied force was recorded as their pain threshold. The test was repeated three times with 60 s of rest between each trial, and the average of the three trials was used for analysis. PPT measurements were taken at baseline, immediately post-exercise, and at 24 and 48 h post-exercise to assess changes in pain perception and recovery dynamics. To minimize inter-rater variability, the same researcher conducted all PPT assessments for a given participant.

### 2.9. Visual Analog Scales

Self-reported measures of muscle soreness, recovery, energy levels, and readiness to train were collected using 100 mm visual analog scales (VASs). Participants marked their responses along a continuum anchored by descriptors at either end of the scale (e.g., “No Soreness” to “Extremely Sore”). These scales were used to quantify participants’ perceived current levels of soreness, recovery, energy levels, and overall readiness to engage in exercise. VASs were collected at baseline, immediately post-exercise, and at 24 and 48 h post-exercise.

### 2.10. Sleep Monitoring

Objective sleep data were collected using a previously validated wearable device (Inspire 2, Fitbit, San Francisco, CA, USA), which participants wore continuously for three consecutive nights leading up to Visits 2, 3, and 4. The devices automatically tracked total sleep duration (in hours) using proprietary accelerometry and photoplethysmography algorithms. This approach has been used previously in similar populations [47]. Subjective sleep quality was assessed each morning using a standardized sleep diary consistent with the Consensus Sleep Diary methodology [48].

### 2.11. Statistical Analysis

Descriptive statistics were calculated using standard statistical methods, with all data in tables presented as means ± standard deviation (SD) and all data in figures presented as mean ± standard deviation (SD). Outlier analysis was conducted by calculating studentized residuals, and changes from baseline scores (deltas) were also assessed for outliers. A threshold of ± 3 SD was used to identify statistical outliers. For missing data, an intent-to-treat approach was employed, with the last observed value carried forward. In cases where baseline values were missing, the median for that variable within the respective group was used as a replacement. For contextual perspective of the magnitude of data replacement that occurred using this approach, less than 2% of all collected data was replaced. For any given variable, no more than two data points were replaced across all study participants across all measured time points.

Outcomes were defined a priori according to the original study objectives. The primary outcomes included changes in force and power production (CMJ, IMTP, isokinetic knee extension), perceived recovery and soreness (VAS and PPT), muscle damage markers (CK and myoglobin), and sprint performance (fastest sprint time, mean split time, and sprint fatigue). Secondary outcomes included anaerobic performance (Wingate peak power and mean power), oxidative stress (MDA), testosterone-to-cortisol ratio, inflammation (TNFα, IL-6, and IL-10), readiness to train, and sleep quality. Tertiary endpoints included CBC, CMP, and reported adverse events. The significance threshold for all hypothesis testing was set at an alpha level of *p* < 0.05. Statistical analyses, including means, standard deviations, normality tests, and independent *t*-tests, Wilcoxon signed-rank tests, and Mann–Whitney U tests were performed using SPSS version 26 (IBM, New York, NY, USA). Graphs were created in R (version 4.4.2; R Foundation for Statistical Computing, Vienna, Austria) using the ggplot2 and patchwork packages. A mixed factorial analysis of variance (ANOVA) model was used to assess all primary, secondary, and tertiary outcomes, including those related to clinical health and safety. When a significant group × time interaction was detected using raw data, the ANOVA model was decomposed by calculating delta scores (change from baseline) and analyzing them using independent *t*-tests. Significant main effects of time were further analyzed using simple main effects. Effect sizes (partial eta squared) on the group × time interaction are reported for the group × time interaction effect. When meaningful baseline differences between groups were identified for select performance outcomes, follow-up analyses of covariance (ANCOVA) were conducted, with the respective baseline value included as a covariate to adjust for between-group differences. This approach was used to evaluate adjusted between-group effects when baseline imbalance was present.

## 3. Results

### 3.1. Study Flow and Participant Characteristics

**Figure 2:**
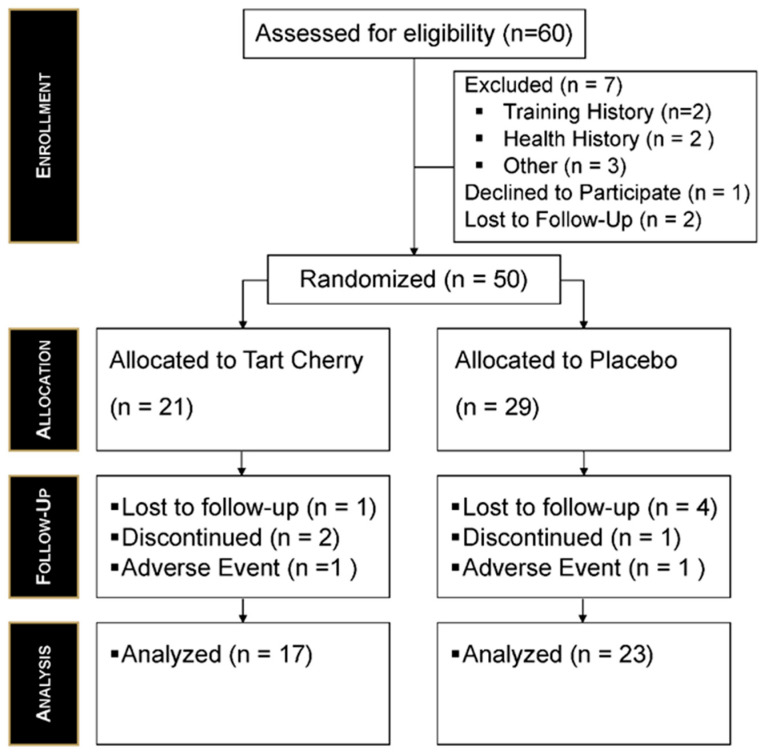
CONSORT diagram outlining participant flow through each phase of the study.

A total of 60 participants were screened for eligibility, with seven excluded (training history: *n* = 2; health history: *n* = 2; other reasons: *n* = 3). A priori sample size estimations were completed using G*Power (version 3.1.9.7). To achieve a small to moderate effect size of 0.20 with an alpha level of 0.05, an assumed power (1 − β) of 0.90, k = 2, four repeated assessments, a total sample size of 46 participants was required. To allow for additional unplanned variability, two additional participants were recruited per group for a total of 50 participants. One participant declined to participate, and two were lost to follow-up before randomization. As such, 50 participants were randomized into the study (TC: *n* = 21; PLA: *n* = 29). Although equal allocation was intended, a logistical error occurred during the distribution of an additional blinded supplement supply near the end of the study. Specifically, the additional capsules provided by the manufacturer were blinded using a coding scheme that differed from the original study codes. As a result, when additional capsules were requested for participants assigned to the TC group, capsules corresponding to the PLA group were inadvertently supplied. This discrepancy was not detected during the study and was only identified after study completion during the unblinding process. Importantly, allocation concealment and blinding of both participants and investigators were maintained throughout the study, and no participants were reassigned or crossed over between conditions in treatment assignment during the distribution of an additional supplement supply late in the study that led to a group imbalance. As a result, 17 participants in the TC group and 23 participants in the PLA group completed the study. Dropouts occurred because of loss to follow-up, discontinuation, and non-study related adverse events. Final analyses included all completing participants (*n* = 40; TC *n* = 17; PLA *n* = 23). The Consolidated Standards of Reporting Trials (CONSORT) diagram for this study is presented in Figure 2. No adverse events were reported to research personnel throughout any part of the study protocol. Additionally, compliance to the supplementation protocol was calculated throughout the study protocol by each study participant. Compliance in the TC group was determined to be 100% (10/10 doses) and PLA 100% (10/10 doses). Independent *t*-tests were used to evaluate homogeneity between the two supplementation groups for age, body mass, and body composition (Table 1).

### 3.2. Countermovement Jump and Isometric Mid-Thigh Pull

**Figure 3:**
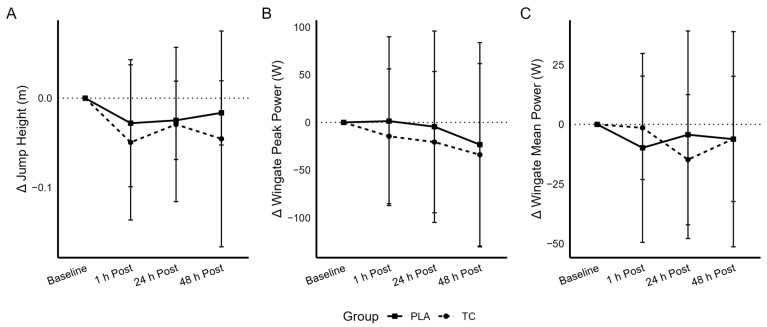
Changes in jump height (**A**), Wingate peak power (**B**), and Wingate mean power (**C**) at each timepoint relative to baseline. The dashed horizontal line represents no change from baseline (Δ = 0).

The group × time interaction for jump height (Figure 3A) was not statistically significant (*p =* 0.608, partial η^2^ = 0.016). A significant reduction in jump height (time effect; *p* = 0.014) was observed, providing evidence of muscle damage. Main effect for group was not significant (*p* = 0.870). No significant group × time interaction was observed for peak propulsive force (*p* = 0.194, partial η^2^ = 0.042). The main effects for time (*p* = 0.146) or group (*p* = 0.365) were similarly not statistically significant. IMTP peak force production indicated no significant group × time interaction (*p* = 0.542, partial η^2^ = 0.018), while the main effect for time (*p* = 0.069) approached statistical significance, and while the group effect (*p* = 0.053) just crossed the statistical significance threshold. IMTP peak force production values were approximately 20% less in the TCR group when compared to PLA at all time points even though these mathematical differences were not statistically different at baseline (independent *t*-test, *p* = 0.097, d = −0.545). These data are observed in Table 2.

### 3.3. Wingate Anaerobic Capacity

Using the Wingate anaerobic capacity test, the following variables were assessed, peak power, mean power, total work, and power production (peak and mean) normalized to body mass, and are provided in Table 2. The group × time interaction for peak power (Figure 3B) was not statistically significant (*p =* 0.928, partial η^2^ = 0.004). The main effect for time was not statistically significant (*p* = 0.193), while the group effect was statistically significant (*p* = 0.020), highlighting greater levels of power production at each measured time point for PLA when viewed against TC. No significant group × time interaction was observed for mean power (Figure 3C) (*p* = 0.410, partial η^2^ = 0.024). The main effect for time (*p* = 0.390) was not statistically significant, while the group effect (*p* = 0.044) was statistically significant. The observed changes in peak power normalized to body mass in kg values were similar (Group × time interaction—*p* = 0.984, partial η^2^ = 0.001), while the main effect for time (*p* = 0.260) was not statistically significant, but the group effect (*p* = 0.011) achieved statistical significance. The group × time interaction for mean power normalized to body mass in kg was not statistically significant (*p =* 0.542, partial η^2^ = 0.017). The main effect for time was not statistically significant (*p* = 0.386), while the group effect was statistically significant (*p* = 0.039). No significant group × time interaction was observed for total work (*p* = 0.366, partial η^2^ = 0.027). The main effect for time (*p* = 0.386) was not statistically significant, while the group effect (*p* = 0.044) was statistically significant. As observed with the statistically significant main effects for group and the observed mathematical differences seen between groups, follow-up independent *t*-tests were performed, which indicated statistically significant differences were observed at baseline (prior to supplementation) for each Wingate variable. Consequently, follow-up analysis of covariance (ANCOVA) was completed with each respective baseline data point being used as a covariate to evaluate between-group differences in each Wingate variable. Using this approach, none of the ANCOVAs approached statistical significance (peak power, *p* = 0.903; mean power, *p* = 0.436; relative peak power, *p* = 0.920; relative mean power, *p* = 0.519; total work, *p* = 0.370).

### 3.4. Isokinetic Dynamometry

As seen in Table 2, peak torque values for the isokinetic knee extension tests were used for data analysis. Peak torque production at 60°·s^−1^ indicated a non-significant group × time interaction (*p =* 0.875, partial η^2^ = 0.003), while the time effect and group effect at this speed were not considered to be statistically significant (time, *p* = 0.093; group, *p* = 0.324). The group × time interaction for peak torque at 180°·s^−1^ was not statistically significant (*p =* 0.335, partial η^2^ = 0.025). The main effect for time was not statistically significant (*p* = 0.119), while the group effect approached statistical significance (*p* = 0.052). No significant group × time interaction was observed for peak knee extension torque at 300°·s^−1^ (*p* = 0.379, partial η^2^ = 0.054), while the main effect for time was deemed to be statistically significant (*p* = 0.027), and the group effect (*p* = 0.054) approached statistical significance.

### 3.5. Repeated Sprints

As completed in previous work by Howatson [43] and Keane et al. [44], the fastest sprint time, average sprint time, and sprint fatigue were calculated. Independent *t*-tests were used to evaluate differences between the groups for these variables. Using this approach, no statistically significant differences were observed between TC and PLA for fastest sprint speed (*p =* 0.160, *d* = 0.459 [ES 95% CI: −0.180–1.091]), average sprint speed (*p =* 0.154, *d* = 0.466 [ES 95% CI: −0.173–1.098]), or sprint fatigue (*p* = 0.814, *d* = 0.076 [ES 95% CI: −0.552–0.702]).

### 3.6. Soreness, Visual Analog Scales, and Pain–Pressure Tolerance

**Figure 4:**
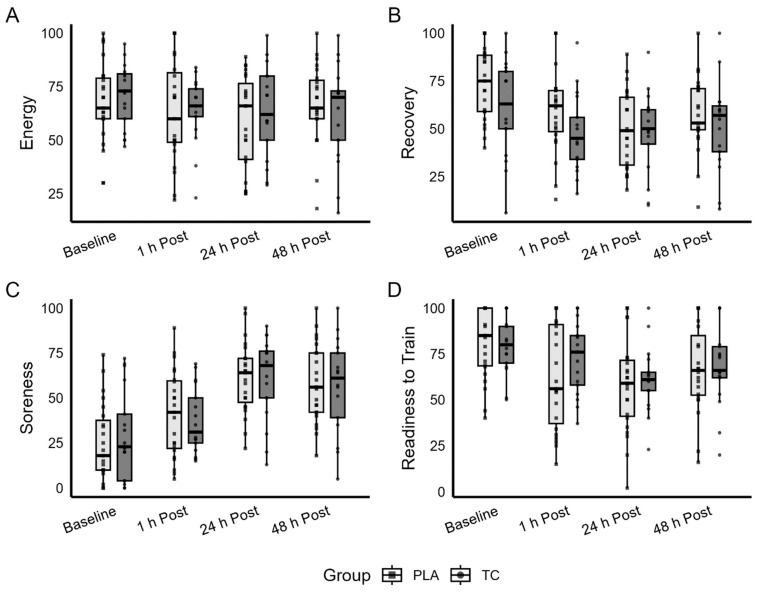
Self-reported energy (**A**), recovery (**B**), soreness (**C**), and readiness to train (**D**) across time. Light gray bars indicate placebo (PLA) and dark gray bars indicate tart cherry (TC).

Visual analog scales 100 mm in length and anchored by consistent terms were completed by all study participants before and in response to the intervention (See Figure 4). Non-significant group × time interactions were observed for energy levels (*p* = 0.398, partial η^2^ = 0.025), recovery (*p* = 0.251, partial η^2^ = 0.035), soreness (*p* = 0.819, partial η^2^ = 0.007), readiness to train (*p* = 0.401, partial η^2^ = 0.025), and PPT (*p* = 0.307, partial η^2^ = 0.031). Significant main effects of time were observed for recovery (*p* < 0.001), soreness (*p* < 0.001), and readiness to train (*p* < 0.001), while energy levels approached significance (*p* = 0.055), providing evidence across both intervention groups of the damaging impact of the sprint protocol. No significant main effects of time were observed for PPT (*p* = 0.383). Main effects of group were not statistically significant for energy (*p* = 0.815), recovery, (*p* = 0.168), soreness (*p* = 0.914), readiness to train (*p* = 0.615), or PPT (*p* = 0.069).

### 3.7. Muscle Damage

As seen in Table 3, creatine kinase and myoglobin changes were evaluated as indicators of muscle damage. Creatine kinase changes indicated a non-significant group × time interaction (*p =* 0.314, partial η^2^ = 0.029), while the main effect of time was statistically significant (*p* = 0.05, partial η^2^ = 0.100), highlighting the anticipated increase in creatine kinase in response to the intervention, while the group effect was not statistically significant (*p* = 0.399). Similarly, myoglobin changes also indicated a non-significant group × time interaction (*p =* 0.499, partial η^2^ = 0.021) and a statistically significant main effect of time (*p* < 0.001, partial η^2^ = 0.363), providing consistent evidence of muscle damage having occurred secondary to the sprint protocol used in this study. The main effect of group for myoglobin was not statistically significant (*p* = 0.836).

### 3.8. Oxidative Stress

As seen in Table 3, malondialdehyde concentrations were evaluated as a marker of oxidative stress. Changes in malondialdehyde indicated a non-significant group × time interaction (*p* = 0.423, partial η^2^ = 0.039), while the main effects of time (*p* = 0.934) and group were not statistically significant (*p* = 0.099). Uric acid, a marker of purine metabolism, did not change in response to the study intervention (group × time, *p =* 0.900, partial η^2^ = 0.004).

### 3.9. Inflammation

TNF-α and IL-6, two predominant cytokines cited in muscle damage literature, were analyzed across the study protocol (Table 3) alongside C-Reactive protein. The group × time interaction for TNF-α (*p* = 0.437, partial η^2^ = 0.036) or IL-6 (*p* = 0.377, partial η^2^ = 0.034) was not statistically significant. Furthermore, the main effects of time (TNF-α: *p* = 0.510; IL-6: *p* = 0.581) and group (TNF-α: *p* = 0.732; IL-6: *p* = 0.926) were not statistically significant for either cytokine. The group × time interaction for C-reactive protein was not statistically significant (*p* = 0.650, partial η^2^ = 0.008), in addition to non-significant main effects for time (*p* = 0.469) and group (*p* = 0.736).

### 3.10. Hormonal Indicators of Recovery or Readiness

Table 3 contains the circulating testosterone and cortisol data as well as the testosterone:cortisol ratio that were evaluated in response to the intervention. No significant group × time interactions were observed for testosterone (*p* = 0.701, partial η^2^ = 0.011), cortisol (*p =* 0.593, partial η^2^ = 0.017), and the testosterone-to-cortisol ratio (*p* = 0.618, partial η^2^ = 0.011). Across the study protocol and as observed through main effects for time, testosterone (*p* = 0.661) and the testosterone-to-cortisol ratio (*p* = 0.164) did not change, while the main effect for time for cortisol was statistically significant (*p* < 0.001).

### 3.11. Sleep Assessment

Significant main effects of time were observed for hours slept (*p =* 0.04), indicating that sleep duration increased from pre- to post-supplementation across both groups. However, there were no significant between-group differences in hours slept (*p* = 0.657). The TC group increased from 6.02 ± 1.26 h at baseline to 6.64 ± 0.90 h post-supplementation (10.2% increase), while the PLA group increased from 6.34 ± 1.23 h to 6.74 ± 0.84 h (6.3% increase). No significant changes in subjective sleep quality were observed within either group across the study period. In the TC group, scores remained stable from Visit 1 (2.94 ± 1.30) to Visit 2 (2.76 ± 1.52; *p =* 0.780, Wilcoxon signed-rank test, *r =* −0.068). Similarly, the PLA group showed no significant change from Visit 1 (3.00 ± 1.38) to Visit 2 (2.48 ± 1.31; *p =* 0.278, *r* = −0.226). Between-group comparisons revealed no significant differences in subjective sleep quality at baseline (*p* = 0.892, *r =* −0.024) or post-supplementation (*p =* 0.588; Mann–Whitney U tests, *r =* −0.092). Device wear compliance was high, with complete objective sleep data obtained for 36 of the 40 participants across the monitoring period. Incomplete recordings in the remaining participants were due to technical or recording issues.

### 3.12. Hematological and Metabolic Markers

Data from complete blood counts and the comprehensive metabolic panels can be found as Table S1 and Table S2, respectively. A significant group × time interaction was found for carbon dioxide levels (*p* = 0.040, partial η^2^ = 0.083); however, all reported carbon dioxide levels remained within clinically accepted normative values for this parameter. Several variables (glucose, creatinine, chloride, calcium, albumin, AST, white blood cell count, and several sub-fraction of white blood cells) demonstrated significant (*p* < 0.05) main effects for time across the study protocol. These outcomes are anticipated due to the intense, damaging nature of the exercise protocol, and the acute time course upon these outcomes was evaluated. Notably, mean changes for these variables remained within clinically accepted normative values for each variable across the study protocol. Beyond that, no significant differences (*p* > 0.05) were observed for any of the other components assessed for either complete blood counts (Table S1) or comprehensive metabolic panels (Table S2).

## 4. Discussion

The findings of this study demonstrate that ten consecutive days of powdered TC supplementation surrounding a bout of maximal sprints in recreationally active men and women did not impact markers of recovery, subsequent bouts of strength and power performance, or sleep when compared to PLA. Due to its potent antioxidant and anti-inflammatory potential, TC consumption has been used for its ability to positively impact recovery and symptoms of muscle damage, thereby helping to increase subsequent exercise performance. A systematic review and meta-analysis by Hill et al. [49] reviewed data from 14 previously published studies for their ability to impact recovery from stressful exercise. TC supplementation was found to have a small beneficial effect in reducing muscle soreness, moderate effects for strength recovery and muscular power, with distinct abilities to restore jumping height while also offering small benefits for sprint time. In terms of candidate biomarkers for inflammation, muscle damage, and oxidative stress, minimal to no benefits were documented in this meta-analysis [49]. Additionally, Gao and Chilibeck 2020 [50] published a meta-analysis that concluded that TC concentrate ingestion could improve endurance exercise performance. Challenging, stressful exercise of varying modality and volume can instigate damage to the underlying muscle tissue, resulting in loss of force and power production potential and a predictable increase in soreness. Further, this structural damage invokes an inflammatory response, which triggers the production of oxidative stress components, which if left unchecked can exacerbate damage to muscle fibers and prolong reductions in strength and power output and increase soreness [51]. TC juice and concentrated powders have increased levels of flavonoids and anthocyanins, key phytonutrients with anti-inflammatory and anti-oxidative potential, which subsequently function, in part, to neutralize these detrimental effects.

Outcomes from the present study contrast with the meta-analytic results of Hill et al. [49] and some of the previous TC supplementation studies that also incorporated other types of high-intensity physical training, and higher-volume or mechanically demanding forms of exercise. While many factors likely converge to explain resulting outcomes from these types of studies, differences in the type and volume of activity employed and training background of the participants take center stage. In this respect, several studies have demonstrated the potential benefits of TC supplementation surrounding high-volume bouts of resistance exercise and other modes of high-volume exercise [4,35,37,52,53,54]. Brown et al. [4] observed that eight days of ingesting Montmorency TC concentrate (30 mL doses, two doses per day) improved recovery of countermovement jump height and tended to reduce soreness in physically active females following a repeated sprint protocol. Similarly, Bell et al. [40] and Quinlan and Hill [41] reported enhanced recovery of force production and attenuated soreness following supplementation with TC concentrate (30 mL doses, two daily doses for 4–6 days) and high-intensity intermittent cycling and sport-specific intermittent sprint exercise, respectively, although effects on biomarkers of muscle damage and inflammation were less consistent. Additionally, studies have been published that utilized powdered TC formulations as in the present study. In this respect, Levers et al. [35] demonstrated that powdered Montmorency TC attenuated performance decrements in resistance-trained males after an intense lower-body workout (10 sets of 10 back squat repetitions with 70% 1RM), though soreness reductions were modest. A poster abstract presented at a conference by Galvan et al. [52] reported cortisol and perceived soreness reductions following a half-marathon run, but performance recovery was not significantly enhanced. Additionally, Hooper et al. [53] had 13 healthy males supplement with either 500 mg of powdered tart cherries or a PLA in a randomized, double-blind, crossover study. A challenging bout of lower-body exercise was completed (six sets of 10 back squat repetitions with 80% 1RM). The supplementation regimen in this study was identical to the present study, whereby each participant supplemented for 7 days prior to completing the exercise bout and continued supplementing each day through 48 h after completing of the exercise bout. These authors reported reductions in oxidative stress and markers of muscle damage along with an attenuation of handgrip strength with no impact on jump height recovery or soreness.

In contrast, Ortega et al. [37] found no improvement in soreness or recovery in a cohort of 17 recreationally active young women following a bout of isokinetic dynamometry using the knee extensors (eight sets of 10 repetitions at 60°/s) and eight days of supplementing with either TC powder or PLA. McCormick and colleagues [54] also reported no changes in performance, soreness, or circulating biomarkers in 12 competitive male water polo players who supplemented with PLA or TC juice for six days prior to completing an in-water water polo performance battery and providing venous blood samples at four timepoints before and after supplementation and the exercise bout. In this study, IL-6 and C-Reactive protein levels did increase after the exercise bout, but these changes were similar between the two supplementation groups. Collectively, these studies highlight the mixed results observed from powdered TC products, with some studies suggesting enhanced recovery [35,52,53], while others [37,54], like the present study, failed to demonstrate benefit. In light of these findings, it is challenging to reconcile the present study’s lack of support for TC supplementation across the diverse outcome domains associated. The exercise bout in the present study was identical to what was employed in the Quinlan and Hill study [41] that demonstrated improvements in force production recovery and soreness, while our supplementation regimen was identical to what was used in the Hooper [53] investigation. It is possible that our mixed sex cohort led to enhanced variability in many of our measured variables, which, when combined with baseline performance disparities between groups, undermined the resulting statistical power. Of the studies highlighted above, the Quinlan and Hill investigation [41] was the only study to use a mixed sex cohort (eight male, 12 female team-sport athletes) who demonstrated improved recovery after completing a modified version of Loughborough Intermittent Shuttle Test. As such, the limited efficacy of TC supplementation observed here may indicate that the mechanisms traditionally associated with recovery, such as reductions in oxidative stress or inflammation, may be less relevant following short-term, high-intensity anaerobic exercise, particularly when mechanical muscle damage is minimal. This thesis has been previously posited by McCormick et al., who also reported no changes in oxidative stress and inflammatory markers as well as no changes in soreness and performance after a similar dosing pattern in well-trained male water polo athletes. Moreover, this suggestion is further supported by the lack of meaningful changes in key performance metrics in the present study, which aligns with previous anaerobic studies that showed inconsistent or negligible effects on strength recovery and soreness [37,54]. Collectively, these findings suggest that the physiological stress induced by repeated sprint activity may not engage the same inflammatory pathways or recovery demands as eccentric-heavy or prolonged endurance protocols, where TC supplementation has demonstrated a more consistent benefit. It should be noted that the repeated sprint exercise was embedded within a broader testing battery of maximal neuromuscular and anaerobic assessments performance before and throughout the recovery period, and therefore the recovery responses observed reflect the cumulative physiological stress of the entire testing protocol rather than the sprint exercise in isolation.

The present study also explored the potential for TC supplementation to impact subjective and objective parameters of sleep. Due to its melatonin content and melatonin’s known role in impacting sleep [55], cherry juice consumption has been proposed as a potential strategy to enhance sleep quality, subsequent recovery, and readiness to train and compete. An initial study by Howatson and colleagues [43] had 20 healthy volunteers complete a randomized, double-blind, crossover design to see if TC juice concentrate consumption for seven days changes urinary production of melatonin as well as indicators of sleep quantity and quality. The authors reported that seven days of consumption significantly increased urinary melatonin content when TC juice was consumed. Additionally, significant increases in time in bed, total sleep time, and sleep efficiency were also reported when TC juice concentrate was consumed, while no changes were observed when PLA was consumed. Additionally, Chung and colleagues [56] supplemented 19 elite female field hockey players with either TC juice or PLA. All participants completed an exhaustive bout of intermittent exercise and subsequently delivered five doses of assigned supplementation for three consecutive days and using actigraphy had sleep parameters assessed. While melatonin and cortisol levels were not impacted, total time in bed, wake time after sleep onset and movement index while sleeping were all significantly improved when TC was consumed. Finally, Hillman et al. [57] supplemented 44 participants for 30 days with either TC or PLA. Four groups were included as half of the cohort took their assigned dose in capsule form while the other half consumed their doses as beverages. Sleep parameters were assessed 0, 14, and 30 days after supplementation. No differences between groups were identified for sleep time or sleep quality, leading the authors to conclude that 30 days of TC consumption in beverage or capsule form did not impact observed changes in sleep behaviors. The present study had all participants wear a wrist-based monitor to assess objective parameters in total sleep along with each participant being asked to provide a subjective assessment of their sleep quality and found that TC supplementation was not responsible for changes in any of the measured sleep parameters within the relatively short supplementation period and observe sleep durations. While consumer-based wearable devices provide practical and ecologically valid estimates of sleep behavior, they rely on proprietary algorithms and do not offer the same level of precision as actigraphy or polysomnography, which should be considered when interpreting these findings. Notably, published data connecting TC supplementation with sleep changes is limited, and of the available literature, discordance appears to exist between these studies. While more research in this area needs to be considered, the two studies which showed positive changes used Actigraphy to evaluate sleep parameters, while the two studies that did not a report a change either used subjective questionnaires or a commercially available wrist-based monitor. Taken together, these findings suggest that any potential sleep-related effects of TC supplementation are likely influenced by supplementation duration, formulation, and baseline sleep characteristics.

An important discussion point for this literature relates to the supplementation type and pattern employed in our study. In terms of pattern, the supplementation protocol employed for this investigation was consistent with other studies [4,40] that also dosed across both pre- and post-exercise windows. However, McHugh [58] previously highlighted that the absolute dose delivered and the form in which it was delivered (juice concentrate, juice from frozen cherries, powdered cherries, etc.) can result in notable differences in the amount of anthocyanins that are delivered as a supplementation. In this respect, the total phenolic content of the TC supplement used in the present study was 1.16% *w*/*w,* resulting in 5.8 mg of phenolic compounds being delivered per dose or 58 mg across the entire supplementation protocol. Disclosure of the actual delivered dose is inconsistently reported in the literature. At present it is unclear if a minimum anthocyanin dose exists to facilitate the cascade of anti-oxidative and anti-inflammatory responses that fosters an environment of heightened recovery. Thus, it remains unclear whether extending the supplementation period would enhance outcomes with powdered products, or whether simply increasing the dose or switching formulations would facilitate more effectiveness on exercise recovery or subsequent performance. As a result, the reader should understand that results from the present study may not fully translate to findings from other TC studies due to differences in dosing, timing, formulation, etc.

With respect to the design limitations of this study, individual variability in response to supplementation likely contributed to the null findings observed in this study. Factors such as baseline fitness levels, training status, sex, and associated sex-related hormonal differences, habitual dietary antioxidant intake, and genetic polymorphisms affecting antioxidant enzyme systems or anthocyanin metabolism may all influence responsiveness. Furthermore, subjects were instructed to maintain consistent dietary intake throughout the study period, yet habitual antioxidant consumption was not quantified or controlled. Additionally, subjects were not directly supervised during their daily supplement consumption, even though they were required to report compliance to the supplementation regimen. To facilitate recreationally active subject pool diversity, activity type, density, history of training, and duration of training were also not specifically monitored before or during the research period beyond the pre-testing restrictions, but again participants were required to not partake in any other activity during the study intervention. Furthermore, hydration status was not directly assessed or standardized across visits, and although participants followed consistent pre-testing instructions, subtle between-visit differences in hydration cannot be ruled out. Finally, the sample size recruited for this study may not have been sufficient to identify small but practically meaningful differences, particularly in the context of a mixed-sex cohort and outcomes with high inter-individual variability, although the sample size recruited for the present study was similar to the other published TC studies. Despite these limitations, this study extends the TC scientific literature to include men and women participating in a repeated sprint protocol. Key strengths include that it is one of a limited number of investigations that use a repeated sprint protocol, an activity with high ecological validity. In addition, the present study compared a battery of outcomes that provided a comprehensive look at recovery considerations from challenging exercise, which included biomarkers related to oxidative stress, inflammation, muscle damage, and anabolic and catabolic hormones while also evaluating perceptual indicators of soreness and readiness, objective indicators of sleep status, and several outcomes related to exercise performance. While no performance or recovery benefits were observed, these findings demonstrate the need for more targeted TC supplementation research surrounding anaerobic, high-speed activity and to better understand if potential benefits are more likely with certain types or volumes of activity. Future research should further explore dose–response relationships, directly compare formulation types, and examine biomarker kinetics in response to varied exercise stressors. No adverse events or supplement-related side effects were reported in this present study, which is consistent with the lack of adverse events reported in other TC supplementation studies [35,52,53].

## 5. Conclusions

In conclusion, under the conditions of the present study, powdered TC supplementation did not heighten recovery or attenuate the observed decrements in exercise performance following repeated sprint exercise in recreationally active adults. However, given the modest sample size, mixed-sex cohort, and associated inter-individual variability, these null findings should be interpreted as context specific. While TC remains a promising intervention in certain exercise contexts, its utility following short-duration repeated sprint exercise may be limited.

## Figures and Tables

**Table 1:** Participant characteristics.

Variable	Group	Mean (SD)	*p*
Age (y)	TC	23.2 (5.3)	0.185
	PLA	25.6 (5.5)	
Height (cm)	TC	170.2 (11.1)	0.581
	PLA	172.2 (10.9)	
Body Mass (kg)	TC	67.9 (12.3)	0.157
	PLA	74.5 (15.6)	
Fat-Free Mass (kg)	TC	30.6 (7.7)	0.181
	PLA	34.2 (8.7)	
Body Fat (%)	TC	20.2 (7.1)	0.508
	PLA	18.6 (7.8)	
BMI (kg·m^−2^)	TC	23.2 (2.2)	0.070
	PLA	24.9 (3.6)	

SD = standard deviation; *p* = *p* value; TC = tart cherry; PLA = placebo; y = year; cm = centimeters; kg = kilograms; m = meters.

**Table 2:** Anaerobic exercise performance.

				Changes from Baseline (Delta)							
Variable	Group	Baseline		1 h Post		24 h Post		48 h Post		(*p*)	
Jump Height (m)	TC	0.311	(0.124)	−0.050	(0.087)	−0.029	(0.087)	−0.046	(0.121)	Time	0.014
	PLA	0.293	(0.093)	−0.028	(0.071)	−0.025	(0.044)	−0.017	(0.036)	G × T	0.608
Peak Propulsive Force (N)	TC	1366	(465)	−31	(92)	19	(134)	64	(199)	Time	0.146
	PLA	1525	(535)	−8	(78)	−6	(123)	−3	(102)	G × T	0.194
IMTP Peak Force (N)	TC	2083	(746)	−105	(318)	−132	(337)	−184	(469)	Time	0.069
	PLA	2489	(745)	−40	(247)	−64	(192)	−53	(217)	G × T	0.542
Wingate Peak Power (W)	TC	576.6	(180.8)	−14.5	(70.6)	−20.6	(74.0)	−33.9	(95.6)	Time	0.193
	PLA	741.7	(262.7)	1.4	(88.5)	−4.5	(100.4)	−23.3	(107.1)	G × T	0.928
Wingate Mean Power (W)	TC	429.4	(130.5)	−1.4	(21.7)	−14.8	(27.3)	−6.1	(26.3)	Time	0.390
	PLA	521.9	(145.0)	−9.8	(39.6)	−4.3	(43.6)	−6.2	(45.2)	G × T	0.410
Wingate Relative Mean Power (W·kg^−1^)	TC	6.12	(1.09)	0.05	(0.49)	−0.15	(0.58)	−0.01	(0.50)	Time	0.386
	PLA	6.99	(1.18)	−0.12	(0.55)	−0.11	(0.60)	−0.09	(0.59)	G × T	0.542
Wingate Relative Peak Power (W·kg^−1^)	TC	8.23	(1.76)	−0.92	(079)	−0.21	(0.93)	−0.38	(1.15)	Time	0.260
	PLA	9.81	(2.04)	0.04	(1.21)	−0.09	(1.46)	−0.26	(1.41)	G × T	0.984
Wingate Total Work (kJ)	TC	12.81	(3.89)	−0.04	(0.65)	−0.45	(0.83)	−0.19	(0.79)	Time	0.394
	PLA	15.56	(4.32)	−0.28	(1.17)	−0.11	(1.29)	−0.99	(1.30)	G × T	0.373
Knee Extension Peak Torque @ 60°·s^−1^(Nm)	TC	154.41	(59.00)	−17.82	(36.31)	−9.74	(77.91)	−22.29	(24.19)	Time	0.093
	PLA	171.91	(59.68)	−21.44	(48.40)	−13.60	(42.39)	−17.54	(41.53)	G × T	0.875
Knee Extension Peak Torque @ 180°·s^−1^ (Nm)	TC	110.50	(46.28)	−3.11	(44.59)	−13.31	(9.21)	−16.51	(26.17)	Time	0.119
	PLA	132.55	(46.48)	−4.19	(14.71)	−3.32	(27.72)	−4.39	(29.87)	G × T	0.335
Knee Extension Peak Torque @ 300°·s^−1^ (Nm)	TC	92.25	(39.87)	−17.81	(31.0)	−12.58	(8.96)	−13.36	(23.17)	Time	0.027
	PLA	107.78	(35.27)	−6.57	(28.9)	−1.37	(18.60)	−4.34	(20.96)	G × T	0.379

Data presented as mean (SD); *p* = *p* value; TC = tart cherry; PLA = placebo; IMTP = isometric mid-thigh pull; m = meters; N = newtons; W = watts; kg = kilogram; KJ = kilojoules; G = group; T = time.

**Table 3:** Blood markers.

				Changes from Baseline (Delta)							
Variable	Group	Baseline		1 h Post		24 h Post		48 h Post		(*p*)	
Creatine Kinase (U∙L^−1^)	TC	180.4	(79.3)	177.5	(78.9)	259.8	(121.4)	195.1	(57.5)	Group (G)	0.399
	PLA	163.9	(52.6)	185.8	(82.7)	383.0	(536.4)	309.5	(444.1)	Time (T)	<0.001
										G × T	0.43
Myoglobin (ng∙mL^−1^)	TC	176.7	(61.8)	708.0	(725.4)	66.3	(109.3)	***	***	Group (G)	0.836
	PLA	175.2	(74.4)	554.9	(592.8)	171.8	(499.7)	***	***	Time (T)	<0.001
										G × T	0.50
Malondialdehyde (µmol∙L^−1^)	TC	1201	(1171)	21.9	(354)	114	(521)	***	***	Group (G)	0.099
	PLA	821	(312)	−24.8	(208)	−78	(369)	***	***	Time (T)	0.934
										G × T	0.42
Testosterone (ng∙dL^−1^)	TC	1.84	(2.27)	−0.014	(0.50)	0.085	(0.60)	0.275	(0.835)	Group (G)	0.395
	PLA	2.31	(1.97)	0.202	(1.31)	0.396	(1.62)	0.127	(2.10)	Time (T)	0.661
										G × T	0.701
Cortisol (µg∙dL^−1^)	TC	276.4	(155.1)	−97.0	(108.9)	−16.5	(68.3)	−18.6	(56.8)	Group (G)	0.304
	PLA	242.6	(149.5)	−95.1	(89.4)	−47.1	(80.7)	−30.2	(74.1)	Time (T)	<0.001
										G × T	0.593
Testosterone:Cortisol Ratio	TC	0.025	(0.067)	0.040	(0.087)	0.038	(0.114)	0.030	(0.081)	Group (G)	0.861
	PLA	0.012	(0.012)	0.037	(0.056)	0.048	(0.109)	0.021	(0.021)	Time (T)	0.164
										G × T	0.618
Uric Acid mg∙dL^−1^)	TC	4.57	(0.79)	1.39	(1.05)	0.65	(0.76)	0.35	(0.95)	Group (G)	0.050
	PLA	5.34	(1.48)	1.57	(1.14)	0.73	(0.79)	0.38	(0.76)	Time (T)	<0.001
										G × T	0.900
C-Reactive Protein (mg∙dL^−1^)	TC	1.13	(1.02)	***	***	0.259	(0.885)	0.072	(0.792)	Group (G)	0.736
	PLA	1.52	(2.56)	***	***	0.065	(0.666)	−0.314	(2.199)	Time (T)	0.469
										G × T	0.650
Interleukin-6 (pg∙mL^−1^)	TC	103.9	(228.5)	−7.81	(61.7)	−24.8	(79.5)	***	***	Group (G)	0.926
	PLA	97.9	(141.2)	0.34	(40.2)	3.5	(48.0)	***	***	Time (T)	0.581
										G × T	0.377
Tumor Necrosis Factor-α (pg∙mL^−1^)	TC	1055	(972)	−172.0	(290.7)	−209.1	(302.6)	***	***	Group (G)	0.732
	PLA	1078	(1202)	28.5	(432.7)	−5.9	(651.4)	***	***	Time (T)	0.47
										G × T	0.44

Data presented as mean (SD); *p* = *p* value; TC = tart cherry; PLA = placebo; *** = data not collected at this timepoint.

## Data Availability

The datasets generated and/or during the current study are available from the corresponding author on reasonable request.

## Supplementary Materials

The following supporting information can be downloaded at https://www.mdpi.com/article/10.3390/nu18030443/s1: Table S1: Comprehensive Metabolic Panel; Table S2: Complete Blood Count with Platelets and Differentials.

## Abbreviations

The following abbreviations are used in this manuscript:

ALT	Alanine aminotransferase
AST	Aspartate aminotransferase
BIA	Bioelectrical impedance analyzer
BMI	Body mass index
BUN	Blood urea nitrogen
CK	Creatine kinase
CMJ	Countermovement jump
DOMS	Delayed-onset muscle soreness
EIMD	Exercise-induced muscle damage
ELISA	Enzyme-linked immunosorbent assay
GFR	Glomerular filtration rate
IPAQ-SF	International Physical Activity Questionnaire—Short Form
IMTP	Isometric mid-thigh pull
IL-6	Interleukin-6
kg	kilograms
kJ	kilojoules
m	meters
MCH	Mean corpuscle hemoglobin
MCHC	Mean corpuscle hemoglobin content
MCV	Mean corpuscle volume
MDA	Malondialdehyde
mL	milliliters
N	Newtons
*p*	Probability level of making Type 1 error
PLA	Placebo
PPT	Pain–pressure tolerance
RDW	Red blood cell dimension width
RSI	Repeated sprint-interval
S	Second
TC	Tart cherry
TNF-α	Tumor necrosis factor-alpha
VAS	Visual analog scale
VL	Vastus lateralis
W	watts

## References

[1] Falvo M.J., Bloomer R.J. (2006). Review of Exercise-Induced Muscle Injury: Relevance for Athletic Populations. Res. Sports Med..

[2] Arazi H., Eston R., Asadi A., Roozbeh B., Saati Zarei A. (2016). Type of Ground Surface during Plyometric Training Affects the Severity of Exercise-Induced Muscle Damage. Sports.

[3] Cleak M.J., Eston R.G. (1992). Muscle Soreness, Swelling, Stiffness and Strength Loss after Intense Eccentric Exercise. Br. J. Sports Med..

[4] Brown M.A., Stevenson E.J., Howatson G. (2019). Montmorency Tart Cherry (*Prunus cerasus* L.) Supplementation Accelerates Recovery from Exercise-induced Muscle Damage in Females. Eur. J. Sport Sci..

[5] Gulick D.T., Kimura I.F. (1996). Delayed Onset Muscle Soreness: What Is It and How Do We Treat It?. J. Sport Rehabil..

[6] Cheung K., Hume P.A., Maxwell L. (2003). Delayed Onset Muscle Soreness: Treatment Strategies and Performance Factors. Sports Med..

[7] Armstrong R.B. (1984). Mechanisms of Exercise-Induced Delayed Onset Muscular Soreness: A Brief Review. Med. Sci. Sports Exerc..

[8] Staublr W.T. (1989). Eccentric Action of Muscles: Physiology, Injury, and Adaptation. Exerc. Sport Sci. Rev..

[9] Smith L.L. (1991). Acute Inflammation: The Underlying Mechanism in Delayed Onset Muscle Soreness?. Med. Sci. Sports Exerc..

[10] Cannon J.G., Blumberg J.B. (2000). Acute Phase Immune Responses in Exercise. Handbook of Oxidants and Antioxidants in Exercise.

[11] Beals K., Allison K.F., Darnell M., Lovalekar M., Baker R., Nieman D.C., Vodovotz Y., Lephart S.M. (2017). The Effects of a Tart Cherry Beverage on Reducing Exercise-Induced Muscle Soreness. IES.

[12] Vider J., Lehtmaa J., Kullisaar T., Vihalemm T., Zilmer K., Kairane Č., Landõr A., Karu T., Zilmer M. (2001). Acute Immune Response in Respect to Exercise-Induced Oxidative Stress. Pathophysiology.

[13] Fallon K.E. (2001). The Acute Phase Response and Exercise: The Ultramarathon as Prototype Exercise. Clin. J. Sport Med..

[14] Mastaloudis A., Morrow J.D., Hopkins D.W., Devaraj S., Traber M.G. (2004). Antioxidant Supplementation Prevents Exercise-Induced Lipid Peroxidation, but Not Inflammation, in Ultramarathon Runners. Free. Radic. Biol. Med..

[15] Urso M.L. (2013). Anti-Inflammatory Interventions and Skeletal Muscle Injury: Benefit or Detriment?. J. Appl. Physiol..

[16] Fridén J., Sjöström M., Ekblom B. (1983). Myofibrillar Damage Following Intense Eccentric Exercise in Man. Int. J. Sports Med..

[17] Lieber R.L., Shah S., Fridén J. (2002). Cytoskeletal Disruption After Eccentric Contraction-Induced Muscle Injury. Clin. Orthop. Relat. Res..

[18] Bowtell J.L., Sumners D.P., Dyer A., Fox P., Mileva K.N. (2011). Montmorency Cherry Juice Reduces Muscle Damage Caused by Intensive Strength Exercise. Med. Sci. Sports Exerc..

[19] Janssen G., Kuipers H., Willems G., Does R.J., Janssen M., Geurten P. (1989). Plasma Activity of Muscle Enzymes: Quantification of Skeletal Muscle Damage and Relationship with Metabolic Variables. Int. J. Sports Med..

[20] Tidball J.G. (1995). Inflammatory Cell Response to Acute Muscle Injury. Med. Sci. Sports Exerc..

[21] Nieman D.C. (1997). Immune Response to Heavy Exertion. J. Appl. Physiol..

[22] Bell P., Walshe I., Davison G., Stevenson E., Howatson G. (2014). Montmorency Cherries Reduce the Oxidative Stress and Inflammatory Responses to Repeated Days High-Intensity Stochastic Cycling. Nutrients.

[23] Powers S.K., Duarte J., Kavazis A.N., Talbert E.E. (2010). Reactive Oxygen Species Are Signalling Molecules for Skeletal Muscle Adaptation. Exp. Physiol..

[24] Connolly D.A.J., McHugh M.P., Padilla-Zakour O.I. (2006). Efficacy of a Tart Cherry Juice Blend in Preventing the Symptoms of Muscle Damage. Br J Sports Med.

[25] Bell P.G., Gaze D.C., Davison G.W., George T.W., Scotter M.J., Howatson G. (2014). Montmorency Tart Cherry (*Prunus cerasus* L.) Concentrate Lowers Uric Acid, Independent of Plasma Cyanidin-3-O-Glucosiderutinoside. J. Funct. Foods.

[26] Bell P.G., McHugh M.P., Stevenson E., Howatson G. (2014). The Role of Cherries in Exercise and Health. Scand. Med. Sci. Sports.

[27] Chandra A., Nair M.G., Iezzoni A.F. (1993). Isolation and Stabilization of Anthocyanins from Tart Cherries (*Prunus cerasus* L.). J. Agric. Food Chem..

[28] Traustadóttir T., Davies S.S., Stock A.A., Su Y., Heward C.B., Roberts L.J., Harman S.M. (2009). Tart Cherry Juice Decreases Oxidative Stress in Healthy Older Men and Women. J. Nutr..

[29] Kelley D.S., Rasooly R., Jacob R.A., Kader A.A., Mackey B.E. (2006). Consumption of Bing Sweet Cherries Lowers Circulating Concentrations of Inflammation Markers in Healthy Men and Women. J. Nutr..

[30] Jacob R.A., Spinozzi G.M., Simon V.A., Kelley D.S., Prior R.L., Hess-Pierce B., Kader A.A. (2003). Consumption of Cherries Lowers Plasma Urate in Healthy Women. J. Nutr..

[31] Zhang Y., Neogi T., Chen C., Chaisson C., Hunter D.J., Choi H.K. (2012). Cherry Consumption and Decreased Risk of Recurrent Gout Attacks. Arthritis Rheum..

[32] Schumacher H.R., Pullman-Mooar S., Gupta S.R., Dinnella J.E., Kim R., McHugh M.P. (2013). Randomized Double-Blind Crossover Study of the Efficacy of a Tart Cherry Juice Blend in Treatment of Osteoarthritis (OA) of the Knee. Osteoarthr. Cartil..

[33] Howatson G., Bell P.G., Tallent J., Middleton B., McHugh M.P., Ellis J. (2012). Effect of Tart Cherry Juice (*Prunus cerasus*) on Melatonin Levels and Enhanced Sleep Quality. Eur. J. Nutr..

[34] Pigeon W.R., Carr M., Gorman C., Perlis M.L. (2010). Effects of a Tart Cherry Juice Beverage on the Sleep of Older Adults with Insomnia: A Pilot Study. J. Med. Food.

[35] Levers K., Dalton R., Galvan E., Goodenough C., O’Connor A., Simbo S., Barringer N., Mertens-Talcott S.U., Rasmussen C., Greenwood M. (2015). Effects of Powdered Montmorency Tart Cherry Supplementation on an Acute Bout of Intense Lower Body Strength Exercise in Resistance Trained Males. J. Int. Soc. Sports Nutr..

[36] Wangdi J.T., O’Leary M.F., Kelly V.G., Jackman S.R., Tang J.C.Y., Dutton J., Bowtell J.L. (2022). Tart Cherry Supplement Enhances Skeletal Muscle Glutathione Peroxidase Expression and Functional Recovery after Muscle Damage. Med. Sci. Sports Exerc..

[37] Ortega D.G., Coburn J.W., Galpin A.J., Costa P.B. (2023). Effects of a Tart Cherry Supplement on Recovery from Exhaustive Exercise. JFMK.

[38] Lamb K.L., Ranchordas M.K., Johnson E., Denning J., Downing F., Lynn A. (2019). No Effect of Tart Cherry Juice or Pomegranate Juice on Recovery from Exercise-Induced Muscle Damage in Non-Resistance Trained Men. Nutrients.

[39] Hillman A.R., Taylor B.C.R., Thompkins D. (2017). The Effects of Tart Cherry Juice with Whey Protein on the Signs and Symptoms of Exercise-Induced Muscle Damage Following Plyometric Exercise. J. Funct. Foods.

[40] Bell P.G., Walshe I.H., Davison G.W., Stevenson E.J., Howatson G. (2015). Recovery Facilitation with Montmorency Cherries Following High-Intensity, Metabolically Challenging Exercise. Appl. Physiol. Nutr. Metab..

[41] Quinlan R., Hill J.A. (2020). The Efficacy of Tart Cherry Juice in Aiding Recovery After Intermittent Exercise. Int. J. Sports Physiol. Perform..

[42] Yu T., Dong K., Jin L. (2024). Effect of Tart Cherry Juice Supplement on Lower Extremity Strength Recovery Performance after Periodization Resisted Sled-Based Training. JOMH.

[43] Howatson G., Milak A. (2009). Exercise-Induced Muscle Damage Following a Bout of Sport Specific Repeated Sprints. J. Strength Cond. Res..

[44] Keane K.M., Salicki R., Goodall S., Thomas K., Howatson G. (2015). Muscle Damage Response in Female Collegiate Athletes After Repeated Sprint Activity. J. Strength Cond. Res..

[45] Comfort P., Dos’Santos T., Beckham G.K., Stone M.H., Guppy S.N., Haff G.G. (2019). Standardization and Methodological Considerations for the Isometric Midthigh Pull. Strength Cond. J..

[46] Blumkaitis J.C., Moon J.M., Ratliff K.M., Stecker R.A., Richmond S.R., Sunderland K.L., Kerksick C.M., Martin J.S., Mumford P.W. (2022). Effects of an External Pneumatic Compression Device vs Static Compression Garment on Peripheral Circulation and Markers of Sports Performance and Recovery. Eur. J. Appl. Physiol..

[47] Kerksick C.M., Moon J.M., Walden K.E., Hagele A.M., Allen L.E., Gaige C.J., Krieger J.M., Jäger R., Pane M., Mumford P. (2024). Multi-Strain Probiotic Improves Subjective Sleep Quality with No Impact on Body Composition, Hemodynamics, and Physical Activity. Benef. Microbes.

[48] Carney C.E., Buysse D.J., Ancoli-Israel S., Edinger J.D., Krystal A.D., Lichstein K.L., Morin C.M. (2012). The Consensus Sleep Diary: Standardizing Prospective Sleep Self-Monitoring. Sleep.

[49] Hill J.A., Keane K.M., Quinlan R., Howatson G. (2021). Tart Cherry Supplementation and Recovery From Strenuous Exercise: A Systematic Review and Meta-Analysis. Int. J. Sport Nutr. Exerc. Metab..

[50] Gao R., Chilibeck P.D. (2020). Effect of Tart Cherry Concentrate on Endurance Exercise Performance: A Meta-Analysis. J. Am. Coll. Nutr..

[51] Harty P.S., Cottet M.L., Malloy J.K., Kerksick C.M. (2019). Nutritional and Supplementation Strategies to Prevent and Attenuate Exercise-Induced Muscle Damage: A Brief Review. Sports Med. Open.

[52] Galvan E., Levers K., Dalton R., Goodenough C., O’Connor A., Simbo S., Barringer N., Carter J., Seesselberg C., Coletta A. (2014). Powdered Tart Cherry Supplementation Effectively Reduces Markers of Catabolism and Perceptions of Muscle Soreness Following an Acute Bout of Intense Endurance Exercise. J. Int. Soc. Sports Nutr..

[53] Hooper D.R., Orange T., Gruber M.T., Darakjian A.A., Conway K.L., Hausenblas H.A. (2021). Broad Spectrum Polyphenol Supplementation from Tart Cherry Extract on Markers of Recovery from Intense Resistance Exercise. J. Int. Soc. Sports Nutr..

[54] McCormick R., Peeling P., Binnie M., Dawson B., Sim M. (2016). Effect of Tart Cherry Juice on Recovery and next Day Performance in Well-Trained Water Polo Players. J. Int. Soc. Sports Nutr..

[55] Pereira N., Naufel M.F., Ribeiro E.B., Tufik S., Hachul H. (2020). Influence of Dietary Sources of Melatonin on Sleep Quality: A Review. J. Food Sci..

[56] Chung J., Choi M., Lee K. (2022). Effects of Short-Term Intake of Montmorency Tart Cherry Juice on Sleep Quality after Intermittent Exercise in Elite Female Field Hockey Players: A Randomized Controlled Trial. Int. J. Environ. Res. Public Health.

[57] Hillman A.R., Trickett O., Brodsky C., Chrismas B. (2022). Montmorency Tart Cherry Supplementation Does Not Impact Sleep, Body Composition, Cellular Health, or Blood Pressure in Healthy Adults. Nutr. Health.

[58] McHugh M.P. (2022). “Precovery” versus Recovery: Understanding the Role of Cherry Juice in Exercise Recovery. Scand. Med. Sci. Sports.

