# Supplementary material for: Impact of Powdered Tart Cherry Supplementation on Performance Recovery Following Repeated Sprint Exercise
Source: Nutrients. 2026 Jan 29;18(3):443. doi: 10.3390/nu18030443 (PMC12899304; doi:10.3390/nu18030443)
Supplement: Supplementary file 1 [file nutrients-18-00443-s001.zip › Table S1.pdf]

Table S1. Comprehensive Metabolic Panel.

		Baseline (Visit 2)	1 h Post (Visit 3)	24 h Post (Visit 4)	48 h Post (Visit 5)	Mixed Factorial ANOVA (*p*)	
**Variable**	**Group**	**Mean (SD)**	**Mean (SD)**	**Mean (SD)**	**Mean (SD)**		
Glucose ($mg \cdot dL^{-1}$)	**TC**	87.94 (11.32)	83.41 (6.04)	87.00 (8.77)	89.82 (11.33)	Group (G)	0.173
	**PLA**	91.04 (7.15)	89.30 (7.65)	89.87 (7.18)	89.52 (5.20)	Time (T)	0.048
						G × T	0.123
BUN ($mg \cdot dL^{-1}$)	**TC**	17.47 (3.79)	16.82 (2.96)	16.88 (4.76)	15.47 (5.60)	Group (G)	0.833
	**PLA**	16.74 (4.78)	16.17 (4.34)	16.30 (4.12)	16.30 (4.74)	Time (T)	0.084
						G × T	0.232
Creatinine ($mg \cdot dL^{-1}$)	**TC**	0.92 (0.16)	0.95 (0.17)	0.88 (0.19)	0.90 (0.18)	Group (G)	0.136
	**PLA**	1.00 (0.19)	1.05 (0.20)	0.97 (0.18)	0.97 (0.19)	Time (T)	<0.001
						G × T	0.897
Sodium ($mmol \cdot L^{-1}$)	**TC**	139.41 (1.42)	138.59 (2.58)	139.00 (2.65)	139.53 (2.40)	Group (G)	0.407
	**PLA**	138.78 (2.26)	138.30 (2.10)	139.04 (1.61)	138.57 (1.78)	Time (T)	0.153
						G × T	0.442
Potassium ($mmol \cdot L^{-1}$)	**TC**	4.36 (0.37)	4.47 (0.43)	4.51 (0.38)	4.41 (0.38)	Group (G)	0.178
	**PLA**	4.34 (0.45)	4.35 (0.29)	4.36 (0.37)	4.24 (0.36)	Time (T)	0.406
						G × T	0.762
Chloride ($mmol \cdot L^{-1}$)	**TC**	104.71 (1.61)	104.12 (2.03)	105.71 (2.11)	105.77 (2.11)	Group (G)	<0.001
	**PLA**	103.09 (1.98)	102.65 (1.94)	104.04 (2.01)	103.74 (2.05)	Time (T)	<0.001
						G × T	0.854
Carbon Dioxide ($mmol \cdot L^{-1}$)	**TC**	26.59 (2.15)	26.12 (2.64)	24.65 (4.11)	25.00 (4.47)	Group (G)	0.156
	**PLA**	26.70 (1.66)	26.74 (1.89)	27.17 (2.64)	26.09 (2.33)	Time (T)	0.051
						G × T	0.040
Calcium ($mg \cdot dL^{-1}$)	**TC**	9.52 (0.33)	9.61 (0.42)	9.36 (0.47)	9.35 (0.38)	Group (G)	0.418
	**PLA**	9.59 (0.41)	9.75 (0.36)	9.45 (0.34)	9.41 (0.41)	Time (T)	<0.001
						G × T	0.781
Albumin ($g \cdot dL^{-1}$)	**TC**	4.51 (0.29)	4.52 (0.27)	4.39 (0.30)	4.42 (0.29)	Group (G)	0.320
	**PLA**	4.60 (0.33)	4.60 (0.29)	4.52 (0.31)	4.50 (0.35)	Time (T)	<0.001
						G × T	0.705
Globulin ($g \cdot dL^{-1}$)	**TC**	2.52 (0.246)	2.48 (0.292)	2.52 (0.307)	2.46 (0.28)	Group (G)	0.851
	**PLA**	2.56 (0.245)	2.54 (0.315)	2.46 (0.291)	2.48 (0.22)	Time (T)	0.222
						G × T	0.310
Albumin: Globulin Ratio	**TC**	1.79 (0.203)	1.82 (0.214)	1.77 (0.229)	1.82 (0.23)	Group (G)	0.600
	**PLA**	1.83 (0.253)	1.84 (0.290)	1.87 (0.304)	1.83 (0.26)	Time (T)	0.824
						G × T	0.344
Bilirubin ($mg \cdot dL^{-1}$)	**TC**	0.659 (0.357)	0.694 (0.368)	0.647 (0.313)	0.635 (0.31)	Group (G)	0.862
	**PLA**	0.617 (0.235)	0.683 (0.267)	0.696 (0.236)	0.700 (0.27)	Time (T)	0.360
						G × T	0.225
AST ($U \cdot L^{-1}$)	**TC**	20.41 (5.58)	21.88 (4.55)	26.88 (7.24)	24.59 (6.07)	Group (G)	0.449
	**PLA**	20.96 (6.53)	22.26 (6.68)	30.57 (17.00)	28.57 (16.63)	Time (T)	<0.001
						G × T	0.387
ALT ($U \cdot L^{-1}$)	**TC**	16.71 (5.95)	16.94 (5.43)	16.24 (5.13)	16.59 (5.20)	Group (G)	0.505
	**PLA**	17.17 (7.90)	17.35 (7.36)	18.30 (8.34)	19.83 (10.72)	Time (T)	0.144
						G × T	0.065

Group × Time = Interaction effect; Time = Main effect for time; Group = Main effect for group; *p* = p value. † = Different than baseline ($p < 0.05$ with Bonferroni adjustments).
