# Supplementary material for: Impact of Powdered Tart Cherry Supplementation on Performance Recovery Following Repeated Sprint Exercise
Source: Nutrients. 2026 Jan 29;18(3):443. doi: 10.3390/nu18030443 (PMC12899304; doi:10.3390/nu18030443)
Supplement: Supplementary file 1 [file nutrients-18-00443-s001.zip › Table S2.pdf]

Table S2. Complete Blood Count with Platelets and Differentials

Variable	Group	Baseline (Visit 2) Mean (SD)	1 h Post (Visit 3) Mean (SD)	24 h Post (Visit 4) Mean (SD)	48 h Post (Visit 5) Mean (SD)	Mixed Factorial ANOVA (*p*)	
White Blood Cell Count (×10^3^·μL^−1^)	**TC**	5.32 (0.95)	5.54 (1.64)	4.97 (1.07)	4.97 (1.14)	Group (G)	0.194
	**PLA**	5.71 (1.33)	6.13 (1.52)	5.39 (1.38)	5.47 (1.43)	Time (T)	0.004
						G × T	0.923
Red Blood Cell Count (×10^6^·μL^−1^)	**TC**	4.70 (.349)	4.82 (.994)	4.50 (.454)	4.62 (.517)	Group (G)	0.167
	**PLA**	5.00 (.656)	4.81 (.495)	4.77 (.537)	4.99 (.934)	Time (T)	0.231
						G × T	0.309
Hemoglobin (g·dL^−1^)	**TC**	14.29 (1.10)	14.58 (2.89)	13.72 (1.41)	14.08 (1.75)	Group (G)	0.400
	**PLA**	14.81 (1.62)	14.30 (1.29)	14.22 (1.49)	14.86 (2.73)	Time (T)	0.287
						G × T	0.394
Hematocrit (%)	**TC**	43.62 (2.66)	44.70 (8.20)	41.84 (3.39)	43.14 (4.95)	Group (G)	0.388
	**PLA**	45.30 (4.86)	43.54 (3.25)	43.46 (3.89)	45.15 (7.70)	Time (T)	0.243
						G × T	0.314
MCH (pg)	**TC**	30.43 (1.69)	30.31 (1.65)	30.51 (1.71)	30.46 (1.68)	Group (G)	0.323
	**PLA**	29.77 (1.66)	29.91 (1.87)	29.94 (1.83)	29.91 (1.86)	Time (T)	0.647
						G × T	0.714
MCHC (g·dL^−1^)	**TC**	32.72 (0.84)	32.55 (1.17)	32.73 (1.27)	32.61 (0.96)	Group (G)	0.654
	**PLA**	32.70 (0.84)	32.84 (0.95)	32.68 (0.82)	32.85 (0.83)	Time (T)	0.987
						G × T	0.454
Mean Corpuscle Volume (fL)	**TC**	92.93 (3.61)	93.13 (4.45)	93.10 (3.76)	93.51 (4.16)	Group (G)	0.098
	**PLA**	91.00 (3.86)	90.91 (4.14)	91.37 (4.39)	90.84 (4.21)	Time (T)	0.738
						G × T	0.405
Red Cell Dimension Width (%)	**TC**	12.64 (0.82)	12.76 (1.30)	12.64 (.698)	12.58 (.738)	Group (G)	0.622
	**PLA**	12.57 (0.57)	12.51 (.640)	12.50 (.634)	12.57 (.723)	Time (T)	0.678
						G × T	0.390
Platelet Count (×10^3^·μL^−1^)	**TC**	238.4 (39.1)	244.7 (56.7)	234.7 (49.5)	238.5 (41.7)	Group (G)	0.851
	**PLA**	243.7 (58.7)	234.3 (55.1)	226.4 (51.2)	240.5 (59.8)	Time (T)	0.310
						G × T	0.489
Mean Platelet Volume (fL)	**TC**	10.75 (0.71)	10.80 (0.78)	10.70 (0.83)	10.82 (0.82)	Group (G)	0.869
	**PLA**	10.65 (0.91)	10.65 (0.91)	10.77 (1.12)	10.80 (1.02)	Time (T)	0.396
						G × T	0.366
Absolute Neutrophils (cells·μL^−1^)	**TC**	2605 (968)	3388 (1618)	2425 (990)	2379 (865)	Group (G)	0.159
	**PLA**	2909 (846)	3907 (1332)	2917 (1166)	2769 (892)	Time (T)	<0.001
						G × T	0.853
Absolute Lymphocytes (cells·μL^−1^)	**TC**	2121 (590)	1644 (487)	1986 (546)	1887 (429)	Group (G)	0.932
	**PLA**	2107 (621)	1572 (535)	1845 (576)	2063 (598)	Time (T)	<0.001
						G × T	0.218
Absolute Monocytes (cells·μL^−1^)	**TC**	445.9 (135.4)	397.9 (140.6)	407.3 (137.2)	400.00 (133.9)	Group (G)	0.737
	**PLA**	455.6 (114.4)	409.1 (97.2)	401.4 (105.0)	432.7 (126.3)	Time (T)	0.003
						G × T	0.586
Absolute Eosinophils (cells·μL^−1^)	**TC**	113.24 (44.75)	73.94 (40.91)	113.47 (52.09)	108.59 (46.40)	Group (G)	0.123
	**PLA**	201.13 (173.51)	140.52 (164.87)	178.13 (209.06)	200.17 (274.96)	Time (T)	0.007
						G × T	0.567
Absolute Basophils (cells·μL^−1^)	**TC**	34.3 (17.5)	34.0 (27.3)	34.4 (21.1)	36.8 (25.7)	Group (G)	0.358
	**PLA**	38.2 (17.9)	43.4 (40.9)	116.5 (390.2)	51.2 (55.4)	Time (T)	0.420
						G × T	0.415

Neutrophils (%)	**TC**	47.8 (11.9)	58.3 (13.0)	47.7 (11.2)	48.5 (10.0)	Group (G)	0.195
	**PLA**	50.1 (7.2)	62.7 (8.3)	53.6 (9.9)	50.3 (8.2)	Time (T)	<0.001
						G × T	0.377
Lymphocytes (%)	**TC**	40.8 (11.7)	32.3 (12.0)	41.2 (11.1)	40.2 (9.8)	Group (G)	0.089
	**PLA**	37.0 (5.7)	27.5 (7.0)	34.9 (8.6)	37.6 (7.0)	Time (T)	<0.001
						G × T	0.470
Monocytes (%)	**TC**	8.44 (2.11)	7.38 (2.26)	8.14 (1.85)	8.29 (2.06)	Group (G)	0.438
	**PLA**	8.15 (1.71)	6.85 (1.43)	7.65 (1.32)	8.00 (1.46)	Time (T)	<0.001
						G × T	0.882
Eosinophils (%)	**TC**	2.135 (0.744)	1.394 (0.688)	2.312 (1.003)	2.259 (0.899)	Group (G)	0.333
	**PLA**	3.005 (2.126)	1.743 (1.632)	2.562 (1.834)	2.481 (1.507)	Time (T)	<0.001
						G × T	0.174
Basophils (%)	**TC**	0.594 (0.321)	0.612 (0.398)	0.688 (0.392)	0.747 (0.458)	Group (G)	0.748
	**PLA**	0.700 (0.325)	0.617 (0.324)	0.709 (0.327)	0.748 (0.354)	Time (T)	0.015
						G × T	0.584

Group × Time = Interaction effect; Time = Main effect for time; Group = Main effect for group; *p* = p value. † = Different than baseline ($p < 0.05$ with Bonferroni adjustments).
